# Injury characteristics and mortality in an emergency department in Ethiopia: a single-center observational study

**DOI:** 10.1186/s12873-024-01017-7

**Published:** 2024-06-07

**Authors:** Helina Bogale Abayneh, Stein Ove Danielsen, Kristin Halvorsen, Stine Engebretsen

**Affiliations:** 1 Department of Nursing and Health Promotion, Faculty of Health Sciences, Oslo Metropolitan University, Oslo, Norway; 2 Department of Emergency and Critical Care Nursing, St Paul Hospital Millennium Medical College, Addis Ababa, Ethiopia

**Keywords:** Injury pattern, Mortality, Pedestrian road traffic injury, Low-income country

## Abstract

**Introduction:**

An injury is described as any damage to the body that impairs health, and its severity can span from mild to life-threatening. On a global scale, injuries account for approximately 4.4 million deaths annually and are anticipated to become the seventh leading cause of death by 2030. In Ethiopia, injuries account for 7% of all deaths, with one of the world's highest rates of road traffic injuries. This study, undertaken at a primary trauma centre in the capital of Ethiopia, aimed to explore the characteristics of injured patients and emergency department mortality as the patient outcome. Understanding the patterns and outcomes of injuries helps to anticipate needs, prioritize patients, and allocate resources effectively.

**Methods:**

A retrospective single-center observational study utilised patient records from September 2020 to August 2021 at Addis Ababa Burn Emergency and Trauma Hospital, located in Ethiopia. A structured checklist facilitated the data collection. All patients arriving in the ED from September 2020 to August 2021 were eligible for the study while incomplete records (missing > 20% of wanted data elements) were excluded.

**Result:**

Of the 3502 injured patients recorded during the study period, 317 were selected. The mean patient age was 30 years, with 78.5% being male. About 8% arrived the emergency department within an hour after the injury. Ambulances transported 38.8% of patients; 58.5% of these were referred from other facilities. The predominant mechanism of injury both in and outside Addis Ababa was pedestrian road traffic injuries (31.4% and 38%). The predominant injury type was fractures (33.8%). The mortality rate was 5%, of which half were pedestrian road traffic incidents.

**Conclusion:**

Pedestrian road traffic injuries were the main cause of injury in and outside of Addis Ababa. A small proportion of patients arrived at the emergency department within the first hour after an injury event. A significant proportion of ambulance-transported patients were referred from other facilities rather than directly from the scene. The overall mortality rate was high, with pedestrian road traffic injury accounting for half of the proportion.

**Supplementary Information:**

The online version contains supplementary material available at 10.1186/s12873-024-01017-7.

## Introduction

An injury is described as any damage to the body that impairs health, and its severity can span from mild to life-threatening [1]. Road traffic injuries, falls, exposure to mechanical forces, fire, heat and hot substances, drowning, interpersonal violence, and self-harm cause approximately 4.4 million deaths per year globally [2]. The World Health Organization (WHO) describes that injuries significantly contribute to the global death and disease burden [1]. Projections suggest that by 2030, injuries will rank as the seventh leading cause of death worldwide [3]. Cumulatively, injuries account for approximately 8% of global deaths and contribute to about 10% of all disability-adjusted life years [2].

The Global status report on road safety, reflecting data from 180 countries, indicates that worldwide the total number of road traffic deaths has plateaued at 1.25 million per year, with the highest road traffic fatality rates in low-income countries [4]. Despite a plan aimed at halving the global number of deaths and injuries from road traffic crashes [4], there has been a significant rise in road traffic injuries in the African region since 2000, with an almost 50% increase in healthy life-years lost [2].

Injuries rank as the third leading cause of death and long-term disability in adults in the Global South [4]. Particularly in Sub-Saharan Africa, injury-related morbidity and mortality rates are alarmingly high, especially in low- and middle-income countries (LMICs) [5]. In Ethiopia, the WHO reports an injury mortality rate of 94 per 100,000 population, contributing to over 8% of the country’s total deaths [6]. A 2020 mortality surveillance in Addis Ababa indicated that injuries accounted for 7% of all fatalities [7]. A study at the University of Gondar referral hospital showed that injuries comprised 25% of all surgical cases [8]. Notably, Ethiopia has among the world’s highest road traffic injury rates, accounting for 79% of fatalities [7].

Although initiation and development of emergency care in public hospitals of Ethiopia commenced in 2007 [9], its infancy is still evident. Initiatives undertaken included the inauguration of the first emergency department (ED), a communication system, and standardisation of drugs, equipment and training [9]. Despite these steps, being a LMIC country, Ethiopia still needs to lay significant groundwork ahead to ensure the quality of public emergency care. This includes well-equipped and adequately staffed EDs and implementation of systematic care protocols [10]. Moreover, there is a pressing need to establish an out-of-hospital emergency care system and provide emergency care training to healthcare providers [10]. The significant injury burden in Ethiopia is exacerbated by inadequate trauma system integration, further underscoring the urgent requirement for enhanced injury care and prevention. Current trauma systems lack the necessary coordination and integration, leading to delays in accessing appropriate care and inefficient use of resources [11].

Hence, conducting research is pivotal in advocating for systematic approaches to enhance emergency care. Understanding the patterns and outcomes of injuries helps emergency department staff provide better and more targeted care. It allows them to anticipate needs, prioritize patients, and allocate resources effectively. Accordingly, the objective of this study was to analyze injury characteristics and patient mortality as an outcome at the Emergency Department (ED) of a tertiary hospital in Ethiopia through a retrospective single-center observational study utilizing patient records.

## Methods

### Study design and setting

We conducted a retrospective single-center observational study at Addis Ababa Burn Emergency and Trauma (AABET) Hospital, located in Addis Ababa in Ethiopia. AABET Hospital is the primary and only trauma and emergency hospital in the country, thus serving a population exceeding 120 million [12]. The hospital provides care for 150,000 patients annually, offering round-the-clock services. The hospital has 11 intensive care unit (ICU) beds and 125 ward beds for neurosurgery, orthopedics, general surgery, plastic and reconstructive surgery for burn patients. Based on the data from 2019, the hospital offers emergency care to over 9500 patients annually, with over 7500 of them being patients with injuries. The ED has three treatment areas based on the triage scale: The Red area with 5 beds, the Orange with 8 beds, and the Yellow and Green with 30 recliners. The ED is staffed with 4 emergency nurses, 68 generic nurses, and 28 emergency physicians.

There is currently no standardised trauma team to manage critically injured patients needing multidisciplinary medical care. The care team is generally assembled post patient arrival and after consultations. Initial patient assessments are performed by the triage team, which then directs to a treatment area or waiting area, based on injury severity. The South African Triage System (SATS) [13] with five triage levels is currently in use. Level 1 indicates an immediate life-threatening condition, Level 2 for an emergency that has the potential to become life-threatening, Level 3 for an urgent situation that is not life-threatening, Level 4 for semi-urgent which doesn’t require immediate response, and Level 5, classified as non-urgent condition but may require treatment.

### Participants and data collection

Eligible patients were all patients arriving in the ED from September 2020 to August 2021. Sample size determination employed a single population proportion formula [14], leveraging data from a prior study at Gondar University Hospital [9], which registered a 25% injury rate. An expected 10% missing due to incomplete (i.e. missing > 20% information) patients’ medical records was integrated. A 95% confidence interval and a 5% margin of error yielded a sample size of 317 from 3502 injured patients during the period of data-collection. The sampling strategy involved choosing a random starting point, then systematically assessing every 11th patient's record, ensuring an unbiased and equitably distributed sample. The Covid 19 pandemic could potentially have resulted in a decrease in the incidence of injuries and visits to EDs. Data were extracted from patients' medical records utilising a structured checklist derived and adapted from the WHO’s injury surveillance guidelines [15] (supplement 1). The modified tool was pre-tested to ensure a complete collection of the pre-determined variables from the records. Fourteen incomplete records (missing over 20% of selected data elements) were excluded. The first author conducted the data extraction.

### Variables

We included age and gender as part of socio-demographic characteristics. Age was used as a continuous variable. Injury location was categorised into two groups: within and outside of Addis Ababa. Time from injury to ED arrival was divided into four categories: less than 1 h, 1–11 h, 12–23 h, and more than 24 h. Patients were classified as either Self-referred or Facility-referred. We distinguished the mode of transportation as ambulance-transported and other methods. Glasgow coma scale was also recorded and grouped as 15 and below 15. Injury severity was assessed using the Revised Trauma Score and classified as Delayed, Urgent, or Immediate [16]. Injury mechanism was categorised as pedestrian road traffic injury (RTI), other type of RTI (car collision and motor cycle), fall, stabbing and sharp cut injury, and other type of injury. Anatomical injury locations on the body were classified as head and spinal cord, chest and abdomen, limb, and polytrauma. Types of injuries were grouped as fracture, pneumothorax, major head injury, minor head injury, pelvic and abdominal injury, poly trauma, and other types of injury. Data concerning initial management were also collected, and included the variables airway management, oxygen delivery, chest decompression, bleeding control, fluid resuscitation, pain management, and immobilisation, all were dichotomous. ED length of stay (LOS) was used as a continuous variable and again categorised into less and more than 24 h. Injury outcome was categorised as alive and dead. Certain variables like comorbidities and specific resuscitation timings were inconsistently available and thus excluded.

### Statistical analysis

Data were entered and checked for input quality using appropriate software validation tool, ensuring the accurate and comprehensive recording of the data. Subsequent analysis was performed using STATA 14. Categorical variables are presented as number and percentage, and continuous variables as mean with confidence interval or median with interquartile range, depending on distribution. In group comparison, the Chi-square test and Fisher's exact test were used for categorical variables. For continuous variables, the t-test was used if the data was normally distributed, and the Mann–Whitney rank sum test if not. All tests were two-sided. Variables with a *p*-value of less than 0.05 were considered statistically significant.

## Result

### Patient and injury characteristics

The study population consisted of 317 patients, mostly males. The origin of injuries was evenly distributed between outside and inside of Addis Ababa and few injured patients arrived hospital within 1 h. More details on patient and injury characteristics, see Table 1.

**Table 1:** Background and injury patterns of injured patients comparing to referral status

	**Whole cohort** ***n*** = 317 (%)	**Self-referred** ***n*** = 110(%)	**Facility referred** ***n*** = 207 (%)
**Age (Mean, CI)**	30 (29.02 – 32.78)	29 (26.7 – 33.06)	31 (29.1 – 33.78)
Male	29 (27.87 – 31.85)	28 (24.95 – 32.35)	30 (28.05 – 32.8)
Female	34 (29.86 – 39.54)	33 (26.64 – 39.41)	36 (28.75 – 43.46)
Male patients	249 (78)	79 (71.8)	170 (82.1) *
Injured outside of Addis Ababa	158 (49.8)	4 (3.6)	154 (74.4) **
Ambulance transported	123 (38.8)	2 (1.8)	121 (58.5) **
Time from injury to ED arrival, hrs, (median, IQR)	4 (10 min – 96)	2 (10 min – 72)	7 (25 min – 96) **
< 1	26 (8.2)	21 (19.1)	5 (2.4)
1–11	196 (61.2)	80 (72.7)	116 (56)
12-n23	25 (7.9)	2 (1.8)	23 (11.1)
≥ 24	63 (19.9)	4 (3.6)	59 (28.5)
GCS scored 15	271 (85.5)	102 (92.7)	169 (81.6) **
Revised trauma score			*
Delayed	261 (82.3)	102 (92.7)	159 (76.8)
Urgent	30 (9.5)	5 (4.5)	25 (12.1)
Immediate	26 (8.2)	3 (2.7)	23 (11.1)
Mechanism of injury			
Pedestrian RTI	110 (34.7)	33 (30)	77 (37.2)
Other type of RTI	32 (10.1)	7 (6.4)	25 (12.1)
Fall	76 (24.0)	34 (30.9)	42 (20.3)
Stabbing and sharp cut	33 (10.4)	12 (10.9)	21 (10.1)
Other	66 (20.8)	24 (21.8)	42 (20.3)
Anatomical site of injury			*
Limb	149 (47.0)	61 (55.5)	88 (42.5)
Head and spinal cord	120 (37.9)	39 (35.5)	81 (39.1)
Chest and Abdomen	33 (10.4)	9 (8.2)	24 (11.6)
Polytrauma	15 (4.7)	1 (0.9)	14 (6.8)

*CI* Confidence interval, *IQR* interquartile range, *ED* Emergency department, *GCS* Glasgow coma scale, *hrs* Hours, *RTI* Road Traffic Injury

**p < 0.001, *p < 0.05

**Fig. 1:**
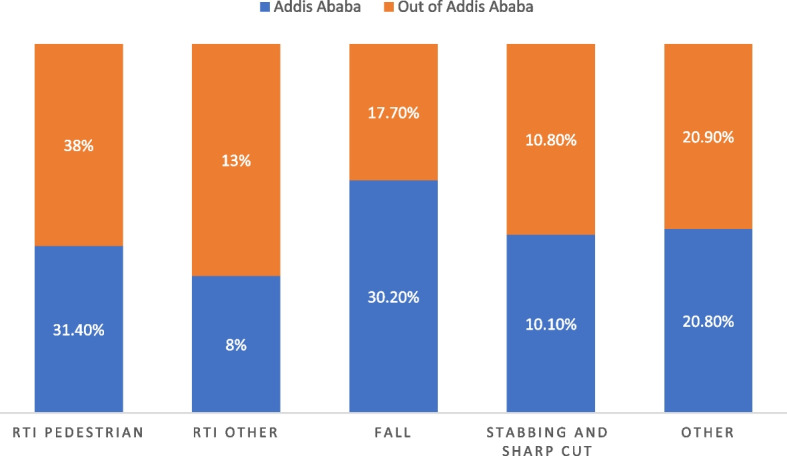
Mechanism of injury based on residence of the study population from AABET hospital

Figure 1 provides an overview of the relation between the mechanisms of injury and where the study population are living. More pedestrian road traffic injuries occurred outside of Addis Ababa and more falling accidents within Addis Ababa, see Fig. 1.

### ED interventions, injury types and mortality

The most common ED management was pain management, and the overall ED mortality was high. More details on ED interventions and mortality, See Table 2.

**Table 2:** Interventions at ED, injury types and mortality of injury comparing with referral status

	**Whole cohort** ***n*** = 317 (%)	**Self-referred** ***n*** = 110 (%)	**Facility referred** ***n*** = 207 (%)
Type of injury			**
Fracture	107 (33.8)	35 (31.8)	72 (35.0)
Pneumothorax	9 (2.8)	1 (0.9)	8 (3.9)
Major head injury	49 (15.5)	7 (6.4)	42 (20.4)
Minor head injury	100 (31.5)	56 (50.9)	44 (21.4)
Pelvic and abdominal injury	13 (4.1)	4 (3.6)	9 (4.4)
Poly trauma	13 (4.1)	1 (0.9)	12 (5.8)
Other	25 (7.9)	6 (5.5)	19 (9.2)
Airway management	36 (11.4)	5 (4.5)	31 (15.0) *
Oxygen delivered	36 (11.4)	5 (4.5)	31 (15.0) *
Chest decompression	8 (2.5)	1 (0.9)	7 (3.4)
Bleeding control	89 (28.1)	30 (27.3)	59 (28.5)
Fluid resuscitation	44 (13.9)	6 (3.5)	38 (18.4) *
Pain management	307 (96.8)	109 (99.1)	198 (95.7)
Immobilization	112 (35.3)	35 (31.8)	77 (37.2)
ED LOS, days (median, IQR)	2 (1—60)		
Length of stay > 24 h	178 (56.2)	39 (35.5)	139 (67.1) **
ED mortality	16 (5.0)	1 (0.9)	15 (7.2) *

*ED* Emergency Department, *IQR* Interquartile range, *LOS* Length of stay, *hrs* Hours

**p < 0.001, *p < 0.05

Of the severely injured patients with an “immediate” trauma severity score at the ED triage, a significantly large number of patients were dead, 87.5% vs 4%, *p* < 0.001. A larger number of injured patients with head or spinal cord injury were dead, 56.2% vs 36.9%, *p* < 0.001, whereas a statistically significant larger proportion of injured patients with polytrauma were dead, 25% vs 3.7%, *p* < 0.001.

### Pedestrian road traffic injury

Pedestrian RTIs were more frequent outside compared to inside Addis Ababa, although not statistically significantly different. More details on pedestrian RTIs, see Table 3

**Table 3:** Injury patterns of patients with pedestrian road traffic injury compared with patients with other injuries

	**Whole cohort** ***n*** = 317 (%)	**Pedestrian road traffic injury** ***n*** = 110 (%)	**Other injuries** ***n*** = 207(%)
Injured in outside of Addis Ababa	159 (50.2)	60 (54.5)	98 (47.3)
Ambulance transported patients	123 (38.8)	55 (50.0)	68 (32.9) *
Facility referred patients	207 (65.7)	77 (70.0)	130 (62.8)
Time from injury to ED arrival, hrs, median, IQR	4 (10 min – 96)	4 (10 min – 86)	4 (10 min – 96)
< 1	26 (8.2)	9 (8.5)	17 (8.3)
1–11	196 (61.2)	70 (66.0)	126 (61.8)
12–23	25 (7.9)	4 (3.8)	21 (10.3)
≥ 24	63 (19.9)	23 (21.7)	40 (19.6)
GCS scored 15	271 (85.5)	89 (80.9)	182 (87.9)
Anatomical site of injury			*
Limb	149 (47.0)	44 (40.0)	105 (50.7)
Head and spinal cord	120 (37.9)	43 (39.1)	77 (37.2)
Chest and Abdomen	33 (10.4)	12 (10.9)	21 (10.1)
Polytrauma	15 (4.7)	11 (10.0)	4 (1.9)
ED LOS > 24 h	178 (56.2)	67 (60.9)	111 (53.6)
ED mortality	16 (5.0)	8 (7.3)	8 (3.9)

ED: Emergency Department, GCS: Glasgow coma scale, hrs.: Hours, IQR: Interquartile range, LOS: length of stay

*p < 0.05

## Discussion

In this study, we aimed to analyse injury characteristics and patient outcomes at a tertiary hospital, and in the following we discuss the main findings related to these aims. Patients mean ED arrival time was 4 h and about 8% arrived within 1 h after the injury occurrence. Pedestrian RTI was the leading mechanism of injury, while fractures and minor head injuries were the leading types of injuries. The median ED length of stay was 2 days, and the overall ED mortality rate was high, 5%.

Our findings are consistent with studies conducted in Africa [17–19]. The elevated percentage of male injuries can be attributed to their gender roles, where the socialisation process leads males to engage in riskier behaviour than females [20]. Men are often involved in greater exposure to outdoor activities, travel risks, and more emotional and risk-taking behaviour compared to females. They are also more likely to work in high-risk jobs and more likely to engage in high-risk sports and activities [20]. The finding that males are more frequently injured may prompt shifts in caregiving dynamics and require adjustments in support systems and safety initiatives within communities. Healthcare policies could prioritize treatments and prevention strategies targeting male-specific injury risks.

It was found that 38.8% of patients received an ambulance service. This percentage is considerably higher compared to a hospital in Zambia (5.8%) [18]. This discrepancy can partially be explained by a high proportion of patients who were transported by an ambulance were from outside of Addis Ababa. Moreover, it is observed that the majority of ambulance-transported patients were facility-referred patients rather than directly from the injury scene. These findings suggest that the high number of ambulance-transported patients from outside of Addis Ababa is not solely due to better ambulance coverage, but due to better ambulance access for transportation between facilities. This indicates that the utilisation of ambulances for injured patient evacuation is still almost negligible, emphasising the need for improvement.

Only 8.2% of patients arrived at the emergency department within one hour, indicating that the majority of victims received limited first-hour trauma treatment at an appropriate trauma centre. This outcome can be attributed to a considerable proportion (65.3%) of patients being referred from other healthcare facilities, which potentially contributed to the delays. Among the patients referred from other facilities, only 2.4% reached the emergency department within the first hour after the injury event, suggesting the possibility that the remaining referred patients received first aid treatment at their initial care centre. Moreover, low ambulance coverage can also be a potential factor. However, most patients in the study cohort were not polytrauma patients.

Based on severity status, a larger proportion of facility-referred patients were identified as “delayed” at ED triage. This finding suggests that these ‘‘delayed’’ patients may have a higher likelihood of receiving treatment at their initial or nearest healthcare facility, potentially contributing to improper patient referrals.

The primary reason for injury occurrence was pedestrian road traffic injury. This contradicts a study from another hospital in Ethiopia, where cuts from sharp tools were reported as the predominant cause of injury [21]. This variation might be attributed to the disparities in study locations, as our research was carried out in the bustling capital city with increased traffic volume. Moreover, the higher proportion of patient referral from inside and outside of the city due to RTIs can also be a potential contributing factor.

Road traffic injuries, which encompassed both pedestrian and other types of road traffic injuries, accounted for 44.7% of all reported injuries. This percentage is higher than the figures reported by hospitals in Ghana, ranging from 14.1% to 39.1%, but similar to the percentages reported by other Ethiopian hospitals, ranging from 47.3% to 49.1%. Nonetheless, it is significantly lower than reported in India, 75% [22–27]. The possible factors for this high number of RTIs could be due to poor conditions of vehicles, poor road systems, non-experienced drivers, poor driving training, over speeding, drunk driving, and improper road use by pedestrians.

The place of injury did not significantly affect the mechanism of injury. However, it was observed that pedestrian road traffic injuries were more prevalent in areas outside of Addis Ababa compared to within the city, despite there being fewer vehicles in those regions compared to the capital Addis Ababa. This might be due to higher speed traffic outside of the city. Providing adequate space for pedestrians, such as well-marked pedestrian crossings, pedestrian islands, and pavements, and reducing the speed limit can enhance pedestrian safety.

ED LOS varied from 1 to 60 days, which was longer compared to the study of Amhara regional hospitals in Ethiopia, where the LOS ranged from 2 to 7 days [28]. The overcrowding and insufficient bed capacity in both the ICU and ward may potentially make patient transferal difficult and result in a prolonged patient stay. Delayed procedures and failure to perform definitive care in a timely manner could potentially contribute to a prolonged stay. It is surprising that more than half of the injured patients remained in the ED for more than 24 h, especially considering that 82.3% of them were classified as “delayed”. These patients should have had the possibility of being discharged within 24 h after receiving mild injury care. Implementing an appropriate and well-communicated patient referral system can prevent improper patient referrals and prolonged ED stays.

The mortality rate was 5%. This finding is higher than the studies reported from Kenya (1.5%), India (1%), Canada (1.2%), Korea (0.6%) and Iran (1.5%), but more align with Sub-Saharan Africa (4.2%) [23, 27, 29–32] and another Ethiopian hospital (6%) [26]. The discrepancies may be attributed to variations in the study samples and areas, prehospital access, differences in the hospitals' readiness for trauma care, and infrastructure.

Extended ED length of stay, without being referred to ICU or respective ward, can contribute to higher mortality [28, 33]. Among injured patients with polytrauma, a significantly larger proportion of patients died in the ED, which can be expected due to higher risk of mortality [34, 35]. Pedestrian road traffic injuries account for half the proportion of mortality compared to both other types of RTIs and all other types of injuries. This indicates that pedestrians are more vulnerable to being injured and killed by an injury than drivers or passengers in vehicles [36, 37].

### Methodological considerations

The study has several strengths. We utilised readily available hospital data records to conduct a comprehensive analysis of injury characteristics, initial managements, and patient outcomes within the trauma centre. This allowed us to draw a valuable conclusion and we are able to recommend improvements based on the results. The findings also serve as inspiration for subsequent studies and further exploration. Additionally, the transparency and clarity exhibited in reporting of methods, research processes, and results contribute to the credibility of the study.

The study also has limitations. Firstly, data was from a single-centre hospital, limiting the external validity. Secondly, the study was retrospective and sensitive to observer bias. Thirdly, there was inaccessible data that could have yielded more explanatory power related to factors such as the specific context of injury and the patients' occupational status. Fourthly, we had no information about treatment initiation time and other care process characteristics. Fifthly, the timing of the study year might have created a selection bias because the data was gathered during the COVID-19 pandemic with "stay-at-home" orders and travel restrictions in place. Finally, the pandemic also reduced injury exposure and injury-related emergency visits, which could have impacted the results in general and the interpretation of the findings.

## Conclusion

Pedestrian road traffic injury was the most common cause of injury, both within and outside of Addis Ababa. The overall ED mortality rate was high, with pedestrian road traffic injuries accounting for half of the rate. Only a small portion of patients reached the ED within the first hour after injury. Most of the patients transported by ambulance were referred from other facilities rather than directly from the scene of injury. There is a need to improve the referral system and prioritize ambulance utilization to reduce time from injury to ED arrival, to ensure a more timely response within the critical first hour. Designing and implementing appropriate prevention strategies against pedestrian road traffic accidents is crucial. Further research on the care processes before and in the ED is warranted, including which factors are associated with mortality.

### Supplementary Information

Supplementary Material 1.

## Data Availability

The datasets used and /or analysed during the current study are available from the corresponding author on reasonable request.

## Abbreviations

AABET: Addis Ababa burn emergency trauma
CI: Confidence interval
ED: Emergency department
ICU: Intensive care unit
LMICs: Low- and middle-income countries
LOS: Length of stay
P: P value
RTA: Road traffic accident
RTI: Road traffic injury
SD: Standard deviation
WHO: World Health Organization
