# Supplementary material for: Injury characteristics and mortality in an emergency department in Ethiopia: a single-center observational study
Source: BMC Emerg Med. 2024 Jun 7;24:97. doi: 10.1186/s12873-024-01017-7 (PMC11157871; doi:10.1186/s12873-024-01017-7)
Supplement: Supplementary file 1 — Supplementary Material 1. [file 12873_2024_1017_MOESM1_ESM.docx]

**Supplement**

1. **Patient demography**

| 1 | Identification code |  | |
| --- | --- | --- | --- |
| 2 | Age | (year) | |
| 3 | Sex | Male |  |
|  |  | Female |  |
| 4 | Place of injury |  | |
| 5 | Referred by | Self-referral |  |
|  |  | Hospital/health center |  |

1. **Trauma characteristics**

| 1 | Mode of transportation | Ambulance | | |  |
| --- | --- | --- | --- | --- | --- |
|  |  | Taxi | | |  |
|  |  | Private vehicle | | |  |
|  |  | Carried | | |  |
|  |  | Other | | | |
| 2 | Trauma time |  | | | |
| 3 | Hospital arrival time |  | | | |
| 4 | place of injury | Addis Ababa | | |  |
|  |  | Outside Addis Ababa | | |  |
| 5 | Mechanism of injury | RTI | | Pedestrian |  |
|  |  |  |  | Car crash (passenger, driver) |  |
|  |  |  |  | Motor cycle |  |
|  |  | Fall | | |  |
|  |  | Assault | | |  |
|  |  | Stab | | |  |
|  |  | Gun shot | | |  |
|  |  | Cut by sharps | | |  |
|  |  | Burn | | | |
|  |  | Other | | |  |
| 6 | Glasgow coma scale at triage |  | | |  |
| 7 | Injury site | Head | | |  |
|  |  | Neck and spinal cord | | |  |
|  |  | Limb | | |  |
|  |  | Abdomen | | |  |
|  |  | Chest | | |  |
|  |  | Poly trauma | | |  |
|  |  | Other | | | |
| 8 | Severity of injury based on revised trauma score (Glasgow coma scale, systolic blood pressure and respiratory rate) | Delayed | | |  |
|  |  | Urgent | | |  |
|  |  | Immediate | | |  |
| 9 | Injury Type | Bruise | | |  |
|  |  | Laceration | | |  |
|  |  | Dislocation | | |  |
|  |  | Fracture | | |  |
|  |  | Amputation | | |  |
|  |  | Chest | Tension pneumothorax | |  |
|  |  |  | Open pneumothorax | |  |
|  |  |  | Hemothorax | |  |
|  |  |  | Lung contusion | |  |
|  |  |  | Other | |  |
|  |  | Abdomen | Blunt | |  |
|  |  |  | Penetrating | |  |
|  |  | Head | Scalp laceration | |  |
|  |  |  | Skull fracture | |  |
|  |  |  | Concussion | |  |
|  |  |  | Epidural hematoma | |  |
|  |  |  | Subdural hematoma | |  |
|  |  |  | Contusion | |  |
|  |  |  | Intracerebral hematoma | |  |
|  |  |  | Other | |  |
|  |  | Pelvic |  | |  |
|  |  | Other | | |  |
| 10 | Intervention in the ED | Airway management | | |  |
|  |  | Oxygen delivery | | |  |
|  |  | Chest decompression | | |  |
|  |  | Bleeding control | | |  |
|  |  | Fluid resuscitation | | |  |
|  |  | Pain management | | |  |
|  |  | Immobilizing fracture | | |  |
|  |  | Other | | |  |
| 11 | Emergency length of stay |  | | |  |

1. **Patient outcome**

| Patient outcome | Live |  |
| --- | --- | --- |
|  | Dead |  |
